# National Neurotrauma Registry Data in Low- and Middle-Income Countries – Current Status and Future Requirements: Comment on "Neurotrauma Surveillance in National Registries of Low- and Middle-Income Countries: A Scoping Review and Comparative Analysis of Data Dictionaries"

**DOI:** 10.34172/ijhpm.2023.7935

**Published:** 2023-05-02

**Authors:** Fiona Lecky

**Affiliations:** CURE, School of Health and Related Research, University of Sheffield, Sheffield, UK

**Keywords:** Registry, Neurotrauma, LMIC

## Abstract

Since 1990 National Trauma Registries, — taking the form of "not for profit" small and medium enterprises — have been integral to improvementsin major injury case fatality in high-income settings. This is laudable but unsatisfactory as globally most years of life lost to injury occur in low- and middle-income countries (LMICs). *International Journal of Health Policy and Management*, recently published a scoping review of neurotrauma registries in LMICs by Barthelemy et al; from this the commentary reflects on the state of the art and how these LMIC registries could be taken to "the next level" as meaningful tools for improving major injury patient care.

 The global burden of injury falls disproportionately on young people living in low- and middle-income countries (LMICs)^1^ and the majority of injury deaths involve neurotrauma (injury to the brain, and or spinal cord).^2^ The recent World Health Assembly resolution illustrates a critical need to understand how healthcare and trauma care systems in LMICs can minimise mortality and disability from RTCs and other major injury vectors — mitigating the unacceptable human cost of “development” (Global Emergency and Trauma Care Initiative; https://www.who.int/news/item/27-05-2019-72nd-world-health-assembly-adopts-resolution-on-emergency-and-trauma-care).

 In recent years hospital case fatality from traumatic brain injury and multisystem injury has been shown to halve in high-income countries (HICs) — associated with improved access to skilled resuscitation and specialist neuroscience care within designated trauma care systems.^3,4^ It has only been possible to demonstrate and publish this evidence with the data acquired, analysed, published and maintained by national trauma registries. Clinicians and Ministries of Health in LMICs — supported by the World Health Organization (WHO) Acute and Trauma Care Programme are keen to see this approach replicated in low resource settings: The current publication by Barthélemy et al “Neurotrauma Surveillance in National Registries of Low- and Middle-Income Countries: A Scoping Review and Comparative Analysis of Data Dictionaries”^5^ provides important insights into how feasible this might be given the “state of the art.”

 Barthélemy and colleagues conducted a scoping review by searching the literature since 1991 for reports of national trauma registries in LMICs where the data dictionaries may be accessible, they also randomly but not comprehensively searched LMIC ministries of health. In total 15 LMICs were identified as having national trauma registries active at some point over the study period with 16 different registries, however only one registry had all the “minimum neurotrauma data” elements of the international registry for trauma and emergency care (IRTEC).^5^ Although the study had limitations particularly around searching it is impossible not to conclude from this review that currently LMIC trauma registries have limited capacity to support neurotrauma care improvement at national and international level — both in terms of their breadth and depth.

 Disease or patient registries are collections of secondary data related to patients with a specific diagnosis, condition, or procedure. Secondary data is extracted from the patient care record rather than requiring new patient contact making them efficient resources for healthcare quality improvement (QI), assurance and comparative effectiveness research. National and international registries are usually anonimised making data analysis ethical when used for governance, service improvement or research. In order to understand why trauma and neurotrauma registries are not ubiquitous in LMICs one must understand their development in HICs over the last 30 years.

 Trauma and neurotrauma registries have been very much a “bottom up” small or medium enterprise and not for profit development in HICs. They are the equivalent of small business “start-ups” which have taken off. The entrepreneurial spirit was born in groups of clinicians, hospital managers, data scientists and patients in Europe, North America, and Australasia. The motivation was the need for data — rather than dictat and dogma — as the primary driver informing QI, governance, research, and national guidelines^6-9^ to improve survival and reduce disability in major injury victims. Investment in Trauma Registry reach and depth has been supported by ministries and healthcare commissioners; witnessing registry potential from published studies in single hospitals or geographical regions. Core neurotrauma data items are relatively sparse 8 in IRTEC and 40 from the Utstein template, recently replicated by the Collaborative European Neurotrauma Effectiveness Research in Traumatic Brain Injury.^10-12^ However, for trauma and neurotrauma registries to “live and breathe” data collection and reporting needs to be continuous and updated. Trauma receiving hospitals need a designated staff member paid to enter data and a clinician supporting this and receiving the registry reports which should benchmark the performance of each hospital against its peers — international and national norms — in a recent data set. The trauma or neurotrauma lead for the registry needs to be responsible for feeding back the reports at hospital trauma audit meetings for learning, governance and QI to occur. A credible mortality risk adjustment model is also key so that hospitals can estimate whether their acute care survival is better or worse than expected, this should be possible with the IRTEC data fields to risk adjust using the Kampala Trauma Score.^13^ Without these feedback loops for QI, governance and learning the registry can wither on the vine and become historical and less relevant like any ageing research dataset.

 The Global Emergency Care research network (GEMCARN) prioritised 7 key questions for improving trauma and emergency care systems. The third highest priority was given to the question *“What are the obstacles to implementing emergency care/trauma registry-based systems in LMICs?”*^14^ Given the history and requirement for a successful neurotrauma registry in resource rich settings one can guess that the barrier is not just a database and/or clinical record which IRTEC has addressed, but the ability to prioritise initiating and maintaining registry based QI and governance in resource limited settings when the actual provision of resuscitation, lifesaving treatment and training clinicians understandably take priority. However, to suggest that “it’s resources” is glib — probably oversimplifying a complex issue. Studies of why data in mass casualty incidents is so limited also suggest it is a cultural issue as much as limited resources.^15^ As with many small and medium enterprises success depends on leadership by key individuals, appetite for change, motivation to succeed — but resources and training in consistent reproducible data collation, reporting and interpretation for QI are also needed. In HICs resources for dedicated registry staff in the co-ordination centre are provided through a variety of funding arrangements from road traffic collision insurance companies, central ministry or surgical college funding or subscriptions from individual hospitals.^6-8^ Trauma registry co-ordination centres are usually situated within higher education institutions rather than as independent entities, and it is worth noting the efficiencies that emanate from an “all major injury” inclusion criterion rather than solely neurotrauma. The rigour of the approach taken by Barthélemy and colleagues suggest the GEMCARN questionshould be given priority by research funders.

## Acknowledgements

 This is an invited commentary after providing review on the *International Journal of Health Policy and Management* scoping review from Barthélemy et al.

## Ethical issues

 Not applicable.

## Competing interests

 Author declares that she has no competing interests.

## Funding

 Global Emergency Care Research Network (GEMCARN)/Lead applicant/GCRF-QR/£50,000/2019-20.
